# CARD9 deficiency improves the recovery of limb ischemia in mice with ambient fine particulate matter exposure

**DOI:** 10.3389/fcvm.2023.1125717

**Published:** 2023-02-13

**Authors:** Qiang Zhu, Xuanyou Liu, Hao Wu, Chunlin Yang, Meifang Wang, Feng Chen, Yuqi Cui, Hong Hao, Michael A. Hill, Zhenguo Liu

**Affiliations:** ^1^Center for Precision Medicine, Division of Cardiovascular Medicine, Department of Medicine, University of Missouri School of Medicine, Columbia, MO, United States; ^2^Dalton Cardiovascular Research Center, University of Missouri, Columbia, MO, United States

**Keywords:** PM exposure, ischemic limb, ROS, oxidative stress, macrophages, CARD9

## Abstract

**Background:**

Exposure to fine particulate matter (PM) is a significant risk for cardiovascular diseases largely due to increased reactive oxygen species (ROS) production and inflammation. Caspase recruitment domain (CARD)9 is critically involved in innate immunity and inflammation. The present study was designed to test the hypothesis that CARD9 signaling is critically involved in PM exposure-induced oxidative stress and impaired recovery of limb ischemia.

**Methods and results:**

Critical limb ischemia (CLI) was created in male wildtype C57BL/6 and age matched CARD9 deficient mice with or without PM (average diameter 2.8 μm) exposure. Mice received intranasal PM exposure for 1 month prior to creation of CLI and continued for the duration of the experiment. Blood flow and mechanical function were evaluated *in vivo* at baseline and days 3, 7, 14, and 21 post CLI. PM exposure significantly increased ROS production, macrophage infiltration, and CARD9 protein expression in ischemic limbs of C57BL/6 mice in association with decreased recovery of blood flow and mechanical function. CARD9 deficiency effectively prevented PM exposure-induced ROS production and macrophage infiltration and preserved the recovery of ischemic limb with increased capillary density. CARD9 deficiency also significantly attenuated PM exposure-induced increase of circulating CD11b^+^/F4/80^+^ macrophages.

**Conclusion:**

The data indicate that CARD9 signaling plays an important role in PM exposure-induced ROS production and impaired limb recovery following ischemia in mice.

## Introduction

Ambient particulate matter (PM) exposure is a significant challenge to public health with significant increase in cardiovascular mortality and morbidity (1). Based on aerodynamic diameter, PM is categorized as coarse particles with a diameter of ≤10 μm (PM10), fine particles with a diameter of ≤2.5 μm (PM2.5), and ultrafine/nanoparticles with a diameter of ≤0.1 μm (PM0.1) (2). Epidemiological studies have shown that increased cardiovascular adverse events are largely related to the exposure to PM2.5 and PM0.1 (2, 3).

Peripheral artery disease (PAD) is an important pathological condition that is frequently associated with significant limb ischemia especially in the patients with diabetes mellitus or hyperlipidemia. Unfortunately, very limited treatment options are available for these patients with less desirable outcome, and amputation is often the treatment of choice. The etiology for PAD is complex, and has not been fully understood. A recent study has shown that PM exposure is associated with a cumulative increase of acute limb ischemia (ALI) hospital admissions (4). PM exposure also attenuates the recovery of limb ischemia in animal studies (5–7). However, the mechanism(s) for the impaired recovery of the ischemic limb is largely undefined.

PM2.5 exposure triggers significant systemic inflammatory responses with increased levels of oxidative stress and reactive oxygen species (ROS) formation with release of large amount of pro-inflammatory cytokines including tumor necrosis factor (TNF)-α, interleukin (IL)-1β, and IL-6 (5, 8). Caspase recruitment domain-containing protein 9 (CARD9) is abundantly expressed in immune cells such as macrophages and dendritic cells and is critically involved in the regulation of immune cell activation and inflammatory responses (9–11). CARD9 functions as an important upstream activator of pro-inflammatory signaling pathways (NF-kB and p38 MAPK signaling) to regulate the productions of a wide spectrum of inflammatory cytokines including TNF-α, IL-1β, and IL-6 (12, 13), thus playing an essential role in ROS production and oxidative stress. It has been reported that CARD9 signaling is involved in PM exposure-induced pulmonary injury (14).

The present study was designed to test the hypothesis that CARD9-mediated signaling is critically involved in PM exposure-induced ROS production and impaired recovery following limb ischemia. There were two objectives: (1) to determine if PM exposure could attenuate the recovery of circulation and mechanical function of ischemic limb in a mouse model; and (2) to define the role of CARD9-mediated signaling in mediating the effect of PM exposure on ROS production and the recovery of ischemic limb.

## Materials and methods

### Animals and PM exposure

All animal studies were performed in compliance with the “Guide for the Care and Use of Laboratory Animals of US National Institutes of Health.” The animal study protocols were reviewed and approved by the Institutional Animal Care and Use Committee of the University of Missouri-Columbia (Protocol #9227). Male wildtype (WT) C57BL/6 mice (8–12 weeks old) and age-matched CARD9 deficient (CARD9^−/−^) mice (Jackson Laboratory, USA) were randomly divided into PM exposure and control groups. PM preparations (Standard Reference Materials 2786) were obtained from the National Institute of Standards and Technology (NIST) with an average diameter of 2.8 μm as described (15). PM was dispersed in PBS (free of endotoxin) by ultrasonication with a concentration of 0.5 μg/μl as detailed in previous publication (16). Each mouse received 10 μg PM (i.e., 5 μg PM in 10 μl for each nostril with a 5 mins interval) *via* intranasal instillation every other day (three times per week) for 4 weeks under general anesthesia with 1.5% isoflurane before the surgery of critical limb ischemia (CLI) with continuation of PM exposure until the end of the experiment, with PBS (free of endotoxin) as the control as described (16, 17).

### Mouse CLI model and evaluations of limb blood flow and mechanical function

There were 4 experimental groups with 7 mice in each group: (1) WT-PBS control (WT C57BL/6 mice with CLI and PBS treatment); (2) WT-PM group (WT C57BL/6 mice with CLI and PM treatment); (3) CARD9-PBS group (CARD9^−/−^ mice with CLI and PBS treatment); and (4) CARD9-PM group (CARD9^−/−^ mice with CLI and PM treatment). For surgical induction of CLI, a 3–5 mm incision was made, with minimal tissue disturbance, and the right femoral artery was identified and ligated using a 6-0 silk suture and then transected, under general anesthesia with isoflurane (1.5%) and constant temperature (37 ± 0.5°C) as described (18, 19). Successful creation of CLI was confirmed by a lack of femoral artery blood flow signal using Laser Doppler perfusion imaging (LDPI, Moor Instruments, Devon, UK). Blood flow recovery of the ischemic limb was evaluated using LDPI as the ratio of blood perfusion in the ischemic (right) limb over the blood perfusion in normal (left) limb before ligation, at 30 mins after ligation and on days 3, 7, 14, and 21 after surgical procedure. LDPI imaging was obtained when the blood flow signal was stable for each mouse at each time point. Recovery of mechanical function of ischemia limb was evaluated through a swimming endurance test and a semi-quantitative assessment of ambulatory impairment and limitation of the ischemic limb (modified clinical standard score) prior to creation of CLI and at 14 and 21 days after ischemic limb surgery as detailed in previous publications (20, 21). The ischemic limb recovery index was determined as following: 0 (flexing the toes to resist gentle traction on the tail), 1 (plantar flexion), 2 (no plantar flexion, but without dragging), and 3 (foot dragging) as described (18, 21).

### CD31 immunofluorescent staining and H&E staining

Gastrocnemius muscle tissue was collected from the ischemic limb. The tissue was weighed, and carefully prepared for immunofluorescent as well as H&E staining at day 21 after ischemia. Multiple cross sections of the muscle were obtained for each muscle sample, and 3 cross sections were randomly chosen from each muscle preparation for each of the histological examinations (CD31^+^ capillary density, H&E staining, CD68^+^/CARD9 double staining, and DHE staining, as detailed below). For CD31 immunofluorescent staining, the frozen sections of 6 μm were fixed with 4% paraformaldehyde for 10 mins after air drying for 15 mins. Sections were subsequently incubated with BSA (2%) for 30 mins at room temperature and the exposed to AF 594 anti-mouse CD31 antibody (Biolegend, 102432) with the dilution factor of 1:400 overnight at 4°C. The preparations were mounted using anti-fading DAPI agent after washing with PBS (3×). Three random fields for each section were imaged with a laser confocal microscope. CD31^+^ capillary density was evaluated quantitatively with ImageJ software. The frozen sections of the ischemic muscle tissue were examined for muscle morphology and structure using a H&E staining kit (Thermo Scientific, Waltham, US) as per manufacturer's protocol. Three independent fields were imaged for each section with an inverted light microscope.

### CD68 and CARD9 immunofluorescent staining and DHE staining

CD68 and CARD9 immunofluorescent staining and dihydroethidium (DHE) staining were performed to evaluate CD68^+^ macrophage numbers, CARD9 fluorescence intensity and ROS production in the ischemic limb, respectively. For CD68 and CARD9 staining, the preparations were incubated with BSA (2%) for 30 mins at room temperature after 10 mins of fixation with paraformaldehyde (4%) and washing thoroughly with PBS (x3). Then, the samples were exposed to Spark YG 570 anti-mouse CD68 antibody (1:500, Biolegend, 137037) at 4°C overnight. For CARD9 staining, the tissue preparations were permeabilized with Triton X-100 (0.3%, Sigma-Aldrich) for 15 mins after washing with PBS (x3), and then incubated with anti-CARD9 antibody (1:500, Biolegend, 679102) overnight at 4°C. After PBS washing (x3), the samples were exposed to AF 488 goat anti-mouse lgG (H+L) secondary antibody (1:1,000, Invitrogen, A11001) for 2 h at room temperature. After three times of PBS washing, the tissue samples were mounted using anti-fading DAPI agent. For both CD68 and CARD9 staining, the corresponding IgG isotype antibodies with a dilution factor of 1:500 were used as negative controls. Three random fields for each section were imaged using a laser confocal microscope. For DHE staining, the preparations were mixed with DHE (1:1,000, Invitrogen, D1168) for 15 mins at room temperature after air drying and fixation. After washing with PBS (x3), the preparations were mounted using anti-fading DAPI agent, and examined using a laser confocal microscope and quantified with ImageJ software.

### Flow cytometric analysis for macrophages and intracellular ROS level

Mouse whole blood was obtained at day 21 after creation of CLI and prepared for flowcytometry analysis with lysis and removal of red blood cells (RBCs) using RBC lysis buffer, as described (22). For macrophage analysis, the CD11b^+^/F4/80^+^ cell population was determined as described (23). Anti-mouse CD11b^+^ PE-Cy5 and anti-mouse F4/80^+^ FITC antibodies were obtained from Biolegend (San Diego, CA, USA). Careful compensation was performed for the cell populations with corresponding isotype antibodies as controls (PE/Cyanine5 IgG2b, κ isotype antibody for CD11b^+^ PE-Cy5; FITC IgG1, κ isotype antibody for F4/80^+^ FITC, from Biolegend). Total cell population was gated, and the macrophage population defined as cells double positive for CD11b^+^/F4/80^+^ as determined by flow cytometry (24). The level of intracellular ROS was quantitatively evaluated with FITC-ROS detection reagents (Invitrogen) as detailed previously (25). Cells were mixed with the reagent at 37°C for 10 mins. After washing with PBS (x3), the labeled cells were suspended in warm PBS, and analyzed with flow cytometry. Fluorescence-positive cells were then quantitatively determined using LSRFortessa X-20 (BD Bioscience, CA) and FlowJo (V10) software.

### Western blot analysis

Gastrocnemius muscle was collected from the ischemic limb and prepared (*via* homogenization and lysis) for western blot analysis as detailed previously (26). The lysates of the muscle tissues were placed on 8% SDS-PAGE gels. After electrophoresis, the preparations were then transferred onto 0.45 μm polyvinylidene difluoride (PVDF) membranes. After blocking with 5% milk in 1 × TBST buffer and incubation with antibody against CARD9 (1:500, Biolegend, 679102) at 4 °C overnight, the PVDF membranes were exposed to the second antibodies for 1 h at room temperature. After brief exposure to electrochemiluminescence (ECL) buffer, the preparations were with an Odyssey Imaging System (Li-Cor Biosciences, Lincoln, NE), and analyzed using ImageJ software.

### Statistical analysis

All the data were presented as means ± standard deviation (SD) and analyzed using GraphPad Prism 8.0 software (San Diego, CA, USA). Two groups of data were analyzed using Student's *t-*test. And multiple groups of data were analyzed using ANOVA followed by Tukey's test for subgroup analysis. The difference was considered statistically significant when a two-tailed *p* value was <0.05.

## Results

### PM exposure significantly attenuated the circulatory and functional recovery of mouse ischemic limb

**Figure 1:**
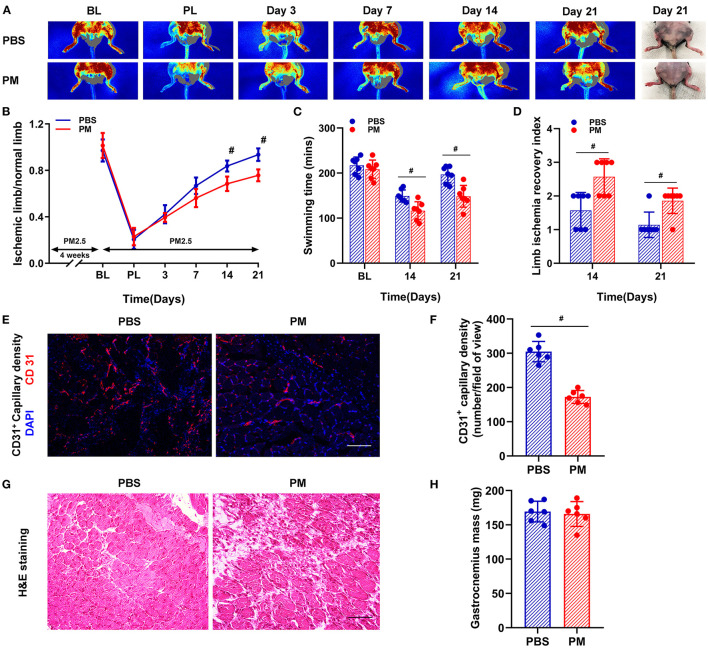
Circulatory and functional recovery of the ischemic limb was attenuated in mice with PM exposure. Recovery of blood flow in the ischemic limb was evaluated by measuring the ratio of ischemic (right) limb blood perfusion/normal (left) limb blood perfusion. PM exposure significantly decreased the recovery of blood flow in C57BL/6 (WT) mice at days 14 and 21 [^#^*p* < 0.01, **(A, B)**]. The mechanical function of the ischemic limb was evaluated using a swimming endurance test and a semi-quantitative assessment of ambulatory impairment and limitation of the ischemic limb (modified clinical standard score). As shown in **(C, D)**, there was a significant decrease in the recovery of mechanical function for mice treated with PM compared to control mice with PBS treatment at days 14 and 21 (^#^*p* < 0.05). Gastrocnemius samples of the ischemic limb was harvested at day 21 after CLI for *ex vivo* histological analyses for capillary density, muscular mass, and inflammatory infiltration. As shown in **(E, F)**, CD31^+^ capillary density was significantly decreased in PM-treated mice compared to PBS-treated mice (^#^*p* < 0.01). H&E staining showed that there was a significant increase in inflammatory cell infiltration in the ischemic limb in PM-treated mice compared to PBS-treated mice, while there was no significant change in muscle mass [^#^*p* > 0.05, **(G, H)**]. BL, before ligation; PL, post ligation; Scale bar, 50 μm. *n* = 7/group. Statistical differences were determined with ANOVA followed by Tukey's post hoc test or Student's two-tailed t-test.

The recovery of blood flow in the ischemic limb was evaluated by measuring the ratio of blood perfusion in the ischemic (right) limb over the blood perfusion in normal (left) limb. As shown in Figures 1A, B, PM exposure significantly decreased the recovery of blood flow in C57BL/6 (WT) mice at days 14 and 21 (PM vs. PBS; 68.4% vs. 83.7% at day 14, and 75.7% vs. 93.6% at day 21, ^#^*p* < 0.01). The mechanical function of ischemic limbs was evaluated using a swimming endurance test and a semi-quantitative assessment of ambulatory impairment and limitation of the ischemic limb (modified clinical standard score). As shown in Figures 1C, D, there was a significant decrease in the recovery of mechanical function for mice treated with PM compared to PBS controls at days 14 and 21 (for swimming time: PM vs. PBS; 116 mins vs. 149 mins at day 14, and 148 mins vs. 197 mins at day 21; for limb ischemia recovery index: PM vs. PBS; 2.6 vs. 1.6 at day 14, and 1.9 vs. 1.1 at day 21; ^#^*p* < 0.05).

*Ex vivo* histological examinations were conducted to determine the capillary density, muscular mass, and inflammatory cell infiltration in the gastrocnemius muscle of the ischemic limb at day 21 after CLI. As demonstrated in Figures 1E, F, CD31^+^ capillary density was significantly decreased in PM-treated mice compared to PBS-treated controls (PM vs. PBS: 172 vs. 305, ^#^*p* < 0.01). The morphology and structure of ischemic muscle were evaluated using H&E staining and muscle mass measurements. There was a significant increase in inflammatory cell infiltration in the ischemic muscle of PM-treated mice compared to PBS-treated controls without significant changes in muscle mass (Figures 1G, H).

### PM exposure increased the levels of ROS production, macrophage infiltration, and CARD9 protein expression in ischemic limbs

**Figure 2:**
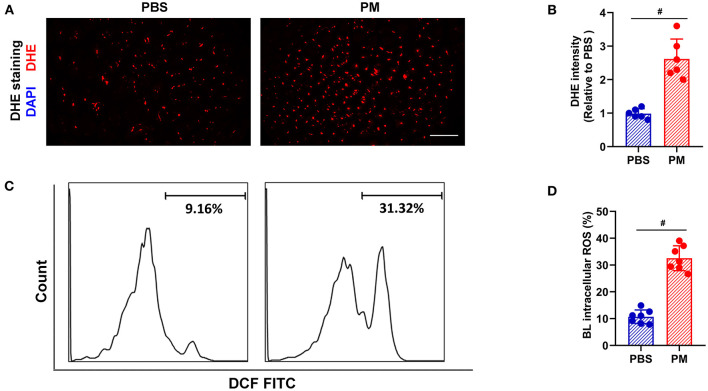
PM treatment increased the levels of ROS in blood and ischemic limb in mice. ROS levels in the ischemic limbs were evaluated using DHE staining, while ROS levels in circulating mononuclear cells were assessed using flow cytometry. As shown in **(A–D)**, the ROS levels in the ischemic tissue and circulating mononuclear cells were significantly increased in PM-treated mice over the controls (^#^*p* < 0.01). DAPI, 4′, 6-diamidino-2-phenylindole; DHE, dihydroethidium; BL, blood; Scale bar, 50 μm. *n* = 6/group. Statistical differences were determined with Student's two-tailed *t*-test.

**Figure 3:**
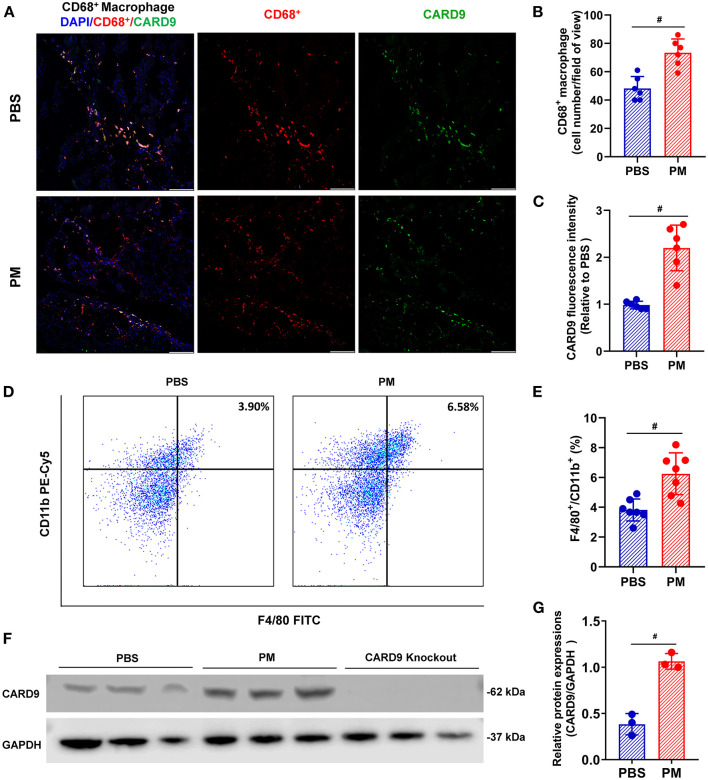
PM exposure increased the numbers of macrophages in blood and ischemic limbs and CARD9 protein level in ischemic limbs. CD68^+^ macrophage infiltration in ischemic limbs was evaluated using immunostaining assay and circulating CD11b^+^/ F4/80^+^ monocytes/macrophages were examined by flow cytometry. As demonstrated in **(A, B, D, E)**, PM treatment significantly increased the levels of CD11b^+^/F4/80^+^ cells in blood and CD68^+^ macrophages infiltration in the ischemic limb compared to PBS-treated control (^#^*p* < 0.01, *n* = 6). Immunostaining and western blot assays showed that CARD9 protein level was significantly increased in the ischemic limb in PM-treated mice compared to PBS-treated controls [^#^*p* < 0.01, *n* = 6, **(A, C, F, G)**]. DAPI, 4,6-diamidino-2-phenylindole; Scale bar, 50 μm. Statistical differences were determined with Student's two-tailed t-test.

Local and circulating levels of intercellular ROS production were evaluated using DHE staining and flow cytometry, respectively. As shown in Figures 2A–D, ROS production in ischemic tissue and circulating monocytes were significantly increased in PM-treated mice (for DHE staining: PM vs. PBS: 2.62 vs. 0.98; for flow cytometry: 32.54% vs. 10.69%, ^#^*p* < 0.01). Similarly, CD68^+^ macrophage infiltration in ischemic limbs was evaluated through immunostaining analysis, and circulating CD11b^+^/F4/80^+^ monocytes were examined using flow cytometry analysis. As demonstrated in Figures 3A–E, PM treatment significantly increased the number of circulating monocytes and regional macrophage infiltration in the ischemic muscle compared to PBS-treated control (for immunostaining: PM vs. PBS: 117.30 vs. 73.50; for flow cytometry: PM vs. PBS: 6.12% vs. 3.85%, ^#^*p* < 0.01). To determine the effect of PM exposure on CARD9 protein expression in ischemic limbs, the western blot and immunostaining assay for CARD9 protein were performed. It was observed that CARD9 expression was significantly enhanced in the ischemic muscle from PM-treated mice compared to PBS-treated controls (for immunostaining staining assay: PM vs. PBS: 94.67 vs. 59.50, ^#^*p* < 0.01, Figures 3A, C; for western blot assay: PM vs. PBS: 1.06 vs. 0.38, ^#^*p* < 0.01, Figures 3F, G).

### CARD9 deficiency effectively prevented PM-induced increase of ROS production and macrophage infiltration

**Figure 4:**
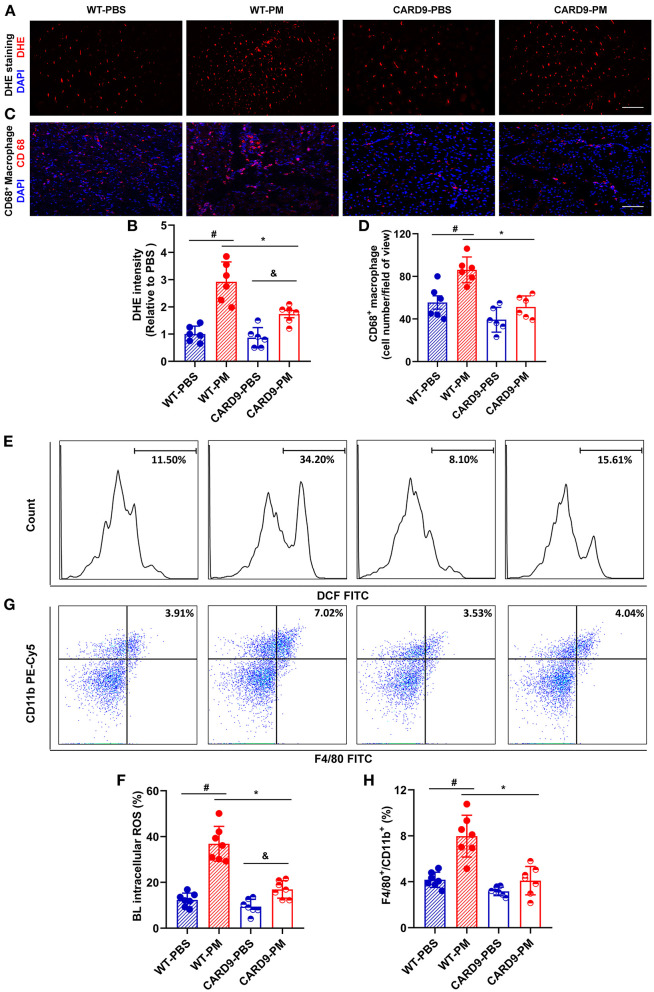
CARD9 deficiency effectively prevented PM-induced increase of ROS production and macrophage infiltration in ischemic limbs. Immunostaining and flow cytometry analysis demonstrated that PM-induced increases in the levels of ROS and macrophages/monocytes in blood and ischemic limbs were significantly attenuated in CARD9 deficient mice [*p < 0.01 for WT-PM vs. CARD9-PM; ^#^*p* < 0.01 for WT-PBS vs. WT-PM; ^&^*p* < 0.05 for CARD9-PBS vs. CARD9-PM, **(A–H)**]. DAPI, 4′,6-diamidino-2-phenylindole; DHE, dihydroethidium; BL, blood; Scale bar, 50 μm. *n* = 6/group. Statistical differences were determined by one-way ANOVA with Tukey's *post-hoc* test.

CARD9 knockout mice were used to repeat the experiments to determine the role of CARD9 signaling in ROS production and macrophage infiltration following PM exposure. Interestingly, PM-induced increases in ROS production (both local and circulating) and macrophage/monocyte infiltration (both local and circulating) were significantly attenuated in CARD9 deficient mice (ROS level using DHE staining: WT-PM vs. CARD9-PM: 2.62 vs. 1.73; ROS level using flow cytometry: WT-PM vs. CARD9-PM: 32.54% vs. 16.92%, ^*^p < 0.01, Figures 4A, B, E, F; macrophage infiltration using immunostaining: WT-PM vs. CARD9-PM: 73.33 vs. 51.33; monocyte cell count using flow cytometry: WT-PM vs. CARD9-PM: 6.12% vs. 4.10%, ^*^p < 0.01, Figures 4C, D, G, H).

### CARD9 deficiency prevented PM-induced impairment in the recovery of the ischemic limb in mice

**Figure 5:**
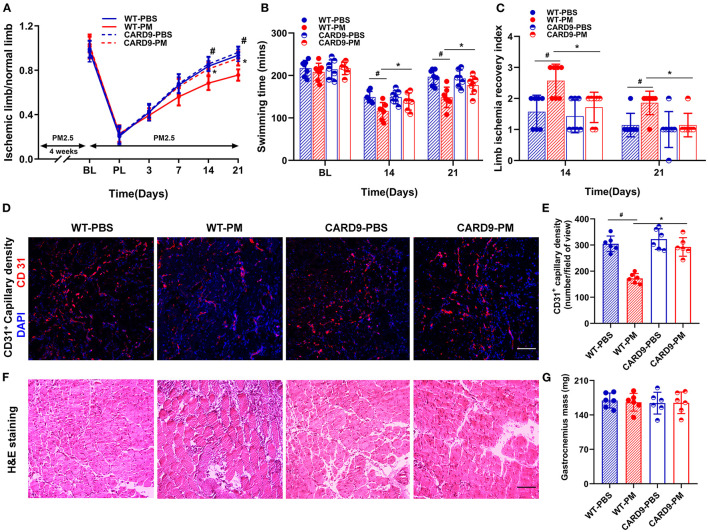
CARD9 deficiency effectively prevented PM-induced impairment in the recovery of blood flow and mechanical function of ischemic limbs, preserved capillary density and attenuated inflammatory infiltration in ischemic limbs. To determine the effect of CARD9 on the recovery of ischemic limb in mice with PM exposure, we performed animal experiment with age-matched CARD9 deficient (CARD9^−/−^) mice. As shown in **(A)**, CARD9 knockout effectively prevented the PM-induced reduction of blood flow recovery (*p < 0.01 for WT-PM vs. CARD9-PM; ^#^*p* < 0.01 for WT-PBS vs. WT-PM). In the meanwhile, CARD9 knockout partially, but significantly reversed the reduction of mechanical function in ischemic limbs in mice with PM exposure at days 14 and 21 [*p < 0.05, ^#^*p* < 0.05; **(B, C)**]. Interestingly, there was no significant difference in the recovery of blood flow and mechanical function between CARD9 and WT mice with PBS treatment. Consistent with the recovery of blood flow and mechanical function, *ex vivo* histological analysis showed that CARD9 deficiency effectively prevented PM-induced decrease of CD31^+^ capillary density and attenuated inflammatory cell infiltration in ischemic limbs [*p < 0.01, ^#^*p* < 0.01; **(D–G)**]. DAPI, 4,6-diamidino-2-phenylindole. Scale bar, 50 μm. *n* = 7/group. Statistical differences were determined with ANOVA followed by Tukey's *post-hoc* test.

To determine the role of CARD9 in PM exposure-induced decreases in blood flow and impairment of mechanical function, experiments were repeated using CARD9^−/−^ mice. As shown in Figure 5A, CARD9 deficiency effectively prevented the PM-induced impairment of blood flow recovery (WT-PM vs. CARD9-PM: 68.4% vs. 81.3% at day 14, and 75.7% vs. 90.7% at day 21, ^*^p < 0.01). In addition, CARD9 deficiency partially, but significantly, reversed the reduction in mechanical function in ischemic limbs of mice exposed to PM at days 14 and 21 (for swimming time: WT-PM vs. CARD9-PM: 116 mins vs. 139 mins at day 21, WT-PM vs. CARD9-PM: 148 mins vs. 177 mins at day 21; for limb ischemia recovery index: WT-PM vs. CARD9-PM: 2.6 vs. 1.7 at day 14, and 1.9 vs. 1.1 at day 21; ^*^p < 0.05, Figures 5B, C). Interestingly, there was no significant difference in the recovery of blood flow and mechanical function between CARD9^−/−^ mice and WT mice without PM exposure. *Ex vivo* histological analyses using CD31 immunofluorescent staining and H&E staining demonstrated that CARD9 deficiency significantly improved CD31^+^ capillary density and decreased inflammatory cell infiltration in ischemic limbs in mice with PM exposure (CD31^+^ capillary density, WT-PM vs. CARD9-PM: 172 vs. 293, ^*^p < 0.01, Figures 5D–G).

## Discussion

In the present study, we demonstrated: (1) exposure to PM2.5 significantly decreased the recovery of blood flow and mechanical function in limb ischemia associated with increased levels of ROS production, macrophage infiltration and CARD9 protein expression; (2) levels of circulating monocytes and their intracellular ROS were significantly increased in PM-treated mice; (3) CARD9 deficiency effectively prevented PM-induced increases of ROS production and macrophage infiltration, while improving circulatory and functional recovery of ischemic limb in mice with PM exposure; and (4) CARD9 deficiency had no impact on ROS production and the recovery of blood flow and function of the ischemic limb in mice without PM exposure. CARD9-mediated signaling was shown to be involved in diet-induced inflammation and cardiac dysfunction as well as metabolic disorders and ischemia/reperfusion cardiac injury (27–29). The present study revealed for the first time that CARD9-mediated ROS production and macrophage infiltration played an important role in the impairment of ischemic limb recovery in mice with PM exposure.

Epidemiological studies have shown that PM exposure increases the risk of CVDs including atherosclerosis, hypertension, arrhythmia, myocardial infarction, and sudden cardiac death (30–33). The mechanisms for increased adverse cardiovascular events associated with PM exposure are complex and clearly multifactorial. However, a common underlying feature that connects PM exposure with CVDs appears to be a significant increase in ROS production and oxidative stress. It is known that PM exposure enhances oxidative stress and inflammatory responses systemically and is associated with various diseases in different organ systems (30, 34, 35). PM exposure-related ROS may come from multiple sources including directly from the PM particles, and more importantly, from various intracellular sources in response to PM exposure through productions of a variety of inflammatory cytokines (8). Inflammatory cytokines promote ROS formation through activation of transmembrane NADPH oxidases (NOXs). ROS in turn stimulates the expressions and releases of pro-inflammatory cytokines through activation of NF-κB signaling pathways (36, 37). We have previously shown that PM exposure enhances ROS levels with increased levels of serum TNF-α, IL-1β, and IL-6 in mice (16, 17). Treatment with Tempol (a SOD mimic) or N-acetylcysteine (NAC) or concomitant overexpression of human superoxide dismutase (SOD)1, SOD3, and glutathione peroxidase (Gpx-1) effectively prevents PM exposure-induced increases of intracellular ROS and serum inflammatory cytokines including TNF-α, IL-1β, and IL-6 in mice (16, 38, 39).

Monocytes and macrophages are an important source of inflammatory cytokines (40, 41). Macrophages play an important role in PM-induced inflammation in respiratory and cardiovascular systems (30, 42). PM exposure significantly enhances inflammatory M1 polarization through ROS-mediated pathway and inhibits anti-inflammatory M2 polarization through a mTOR-dependent mechanism (43). Further, PM exposure has been shown to increase the release of pro-inflammatory mediators including TNF-α, IL-6, and granulocyte-macrophage colony-stimulating factor (GM-CSF) from lung macrophages into circulation (43, 44). It has also been reported that acute exposure to PM induces a sustained activation of macrophages in lung, and enhances leukocyte rolling, adhesion, and transmigration, and aggravates experimental myocardial infarction with an increased infarction size (44). Long-term PM exposure increases macrophage recruitment and lipid content and decreases fibrous cap thickness and SMCs infiltration in atherosclerotic plaques in HFD-fed mice (45). In the present study, we observed that PM exposure significantly attenuated the recovery of limb ischemia in mice in association with increased numbers of circulating monocytes and infiltration of macrophages into the ischemic muscle. The findings of increased numbers of circulating monocytes and infiltrated macrophages in the ischemic areas are consistent with significant increases in intracellular ROS level in the circulating monocytes and tissue ROS levels in the ischemia muscle in mice exposed to PM. However, how PM exposure could increase macrophage infiltration and ROS production in the ischemic muscle is unclear. It is certainly possible that PM exposure-induced productions of inflammatory cytokines including TNF-α, IL-1β, and IL-6 may significantly contribute to monocyte recruitment to and/or macrophage proliferation into the ischemic area. Consistent with this, cytokines from alveolar macrophages and bronchial epithelial cells may stimulate bone marrow with resultant leukocytosis and activate vascular endothelial cells (41). In addition, cell surface adhesion molecules including intercellular adhesion molecule 1 (ICAM-1) and vascular cell adhesion protein 1 (VCAM-1) (46, 47), as well as inflammatory cytokine expression (41) are upregulated in endothelium in response to ischemic injury. These adhesion proteins including their soluble forms are important for recruiting monocytes and lymphocytes into circulation and ischemic tissues (41, 48, 49).

Limb ischemia is well known to increase ROS production both systematically and locally in the ischemic area (50, 51). In the present study, we showed that ischemia-induced ROS production was further increased in mice with PM exposure. Importantly, we also demonstrated that PM exposure significantly increased macrophage infiltration/accumulation in the ischemic muscle and the expression of CARD9 in the ischemic tissue with increased ROS production. CARD9 deficiency had no impact on ischemia-induced macrophage infiltration/accumulation and ROS production in mice without PM exposure. Interestingly, CARD9 deficiency effectively prevented PM exposure-induced enhancement of macrophage infiltration/accumulation and ROS production in the ischemic muscle following PM exposure. These data suggest that PM exposure-induced expression of CARD9 might be critical to ROS production and monocyte/macrophage infiltration in ischemic limbs. However, the mechanism(s) for PM exposure-induced increase in CARD9 expression in the macrophages in the ischemic limb of mice is unclear at this point. It is known that ROS in the lungs enhances the signal transduction of pattern recognition (e.g., TLRs) (52–54), thus increasing the expression of a variety of inflammatory cytokines and chemokines (8). CARD9 is a crucial molecule that mediate the signaling of TLRs and the activations of MAPK and NF-κB, leading to the productions of many important cytokines including (but not limited to) TNF-α, IL-6, IL-2, IL-12p40 (9, 55). Studies have shown that CARD9 signaling is critically involved in diet-induced myocardial dysfunction (27, 56), and obesity-associated metabolic disorders (28). The data from the present study demonstrated that CARD9 signaling was critically involved in ROS production and macrophage infiltration following PM exposure. CARD9-mediated oxidative stress is an important mechanism for the impaired recovery of ischemic limb in mice with PM exposure.

PM exposure triggers a significant systemic inflammation with increased levels of oxidative stress and inflammatory cytokines including TNF-α, IL-6, and IL-1β. It is known that CARD9 is expressed in various types of cells with different functions including macrophages, neutrophils, dendritic cells, lymphoid cells, endothelial cells, cardiomyocytes, and microglial cells (29). However, CARD9 is predominantly expressed in immunoreactive cells especially macrophages and dendritic cells, and is critically involved in the productions of a wide range of cytokines (TNF-α, IL-6, and IL-1β) and chemokines (CXCL1, CXCL2, and CXCL8), which are primarily associated with local and systemic inflammation, oxidative stress, and the development and progression of a variety of diseases including cardiovascular diseases and cancers (57–59). Although many cells can produce inflammatory cytokines, macrophages are an important source for inflammatory cytokines in response to PM exposure (30, 42). In the present study, we observed that PM exposure significantly increased the numbers of circulating monocytes and infiltration of macrophages in the ischemic limb muscle of mice with increased CARD9 expression and ROS production. CARD9 deficiency effectively prevented PM exposure-induced macrophage infiltration/accumulation and ROS production in the ischemic muscle. Interestingly, CARD9 deficiency had no impact on ROS production and the recovery of blood flow and function of the ischemic limb in mice without PM exposure. These data suggest that CARD9-mediated signaling is essential for the development of inflammation and ROS production in the ischemic limb in response to PM exposure in mice. However, further studies are needed to determine if macrophages or other specific cells (e.g., neutrophils or dendritic cells, or endothelial cells) play a dominant role in PM exposure-induced ROS production and impairment of ischemic limb recovery. Future studies are also needed to determine which inflammatory cytokine(s) contributes critically to PM exposure-induced ROS production and inflammation in the ischemic limb in mice.

There were some other limitations in the present study, including (1) No detailed studies to define the complex, and yet critical roles or mechanisms of CARD9-mediated signaling in the pathophysiology of CLI in mice with PM exposure; and (2) No studies to determine if a significant sex difference in the recovery of CLI in mice with PM exposure. There are substantial sex differences in many cardiovascular diseases without well-defined mechanisms. Recently, we observed that there was a significant sex difference in the levels of serum inflammatory cytokines including TNF-α, IL-1β, and IL-6 as well as circulating endothelial progenitor cells (EPCs) in mice after PM exposure (17). The levels of serum inflammatory cytokines especially TNF-α was significantly lower in female mice with PM exposure than that in age-matched males with preserved level of circulating EPCs independent of female sex hormone estrogen (17). EPCs are involved in angiogenesis and ischemic limb recovery, thus, it is important to determine if there are significant sex differences in CARD9 expression, ROS production, macrophage infiltration, and the recovery of CLI in mice with PM exposure in future studies.

In conclusion, the present study demonstrated that PM exposure significantly decreased the recovery of blood flow and mechanical function in ischemic limbs with increased ROS production, and monocyte/macrophage infiltration through a CARD9-mediated mechanism. The data may provide a potential novel target or strategy for CLI patients with refractory limb ischemia with exposure to PM.

## Data availability statement

The original contributions presented in the study are included in the article/supplementary material, further inquiries can be directed to the corresponding author.

## Ethics statement

The animal study was reviewed and approved by the Institutional Animal Care and Use Committee of the University of Missouri-Columbia.

## Author contributions

ZL and QZ contributed to the study conception and design. QZ, XL, HW, CY, MW, and FC performed the experiments and collected the data. QZ, YC, and HH did the statistical analysis. QZ drafted the manuscript. QZ, MH, and ZL critically reviewed the data and revised the manuscript. ZL supervised the study and provided financial supports. All authors carefully reviewed the manuscript and agreed on publication of the data.
